# Supplementary material for: Association between oral microbiome and seven types of cancers in East Asian population: a two-sample Mendelian randomization analysis
Source: Front Mol Biosci. 2023 Nov 21;10:1327893. doi: 10.3389/fmolb.2023.1327893 (PMC10702768; doi:10.3389/fmolb.2023.1327893)
Supplement: Supplementary file 1 [file DataSheet1.ZIP › Supplementary Materials/MR plots of saliva/saliva═╝/Prostate cancer/pheno.1234_to_prostate cancer_leave_one_out.pdf]

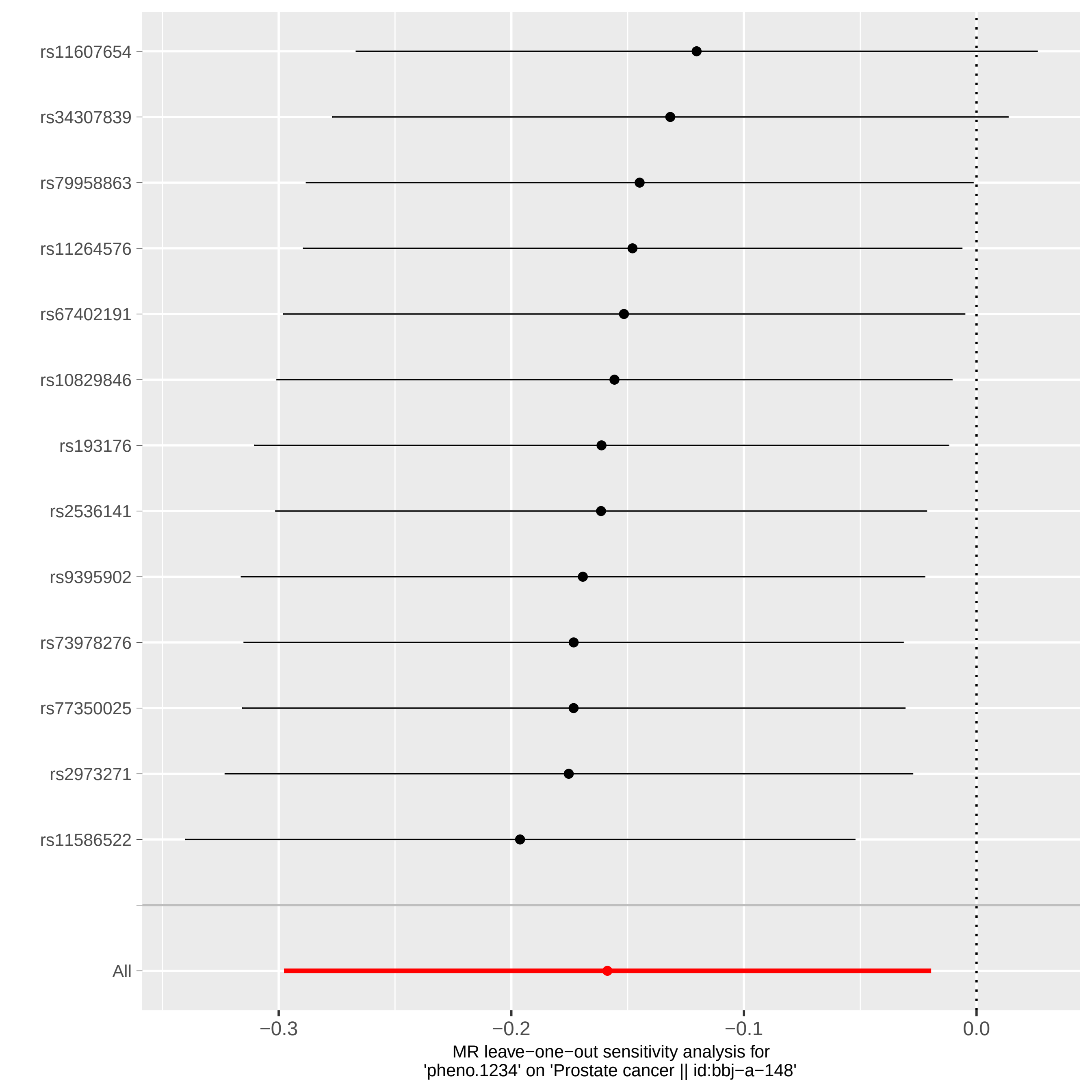

rs11607654
rs34307839
rs79958863
rs11264576
rs67402191
rs10829846
rs193176
rs2536141
rs9395902
rs73978276
rs77350025
rs2973271
rs11586522
All
−0.3
−0.2
−0.1
0.0
MR leave−one−out sensitivity analysis for
'pheno.1234' on 'Prostate cancer || id:bbj−a−148'
